# P47^phox−/−^ Mice Are Compromised in Expansion and Activation of CD8^+^ T Cells and Susceptible to *Trypanosoma cruzi* Infection

**DOI:** 10.1371/journal.ppat.1004516

**Published:** 2014-12-04

**Authors:** Monisha Dhiman, Nisha Jain Garg

**Affiliations:** 1 Department of Microbiology and Immunology, University of Texas Medical Branch (UTMB), Galveston, Texas, United States of America; 2 Department of Pathology, UTMB, Galveston, Texas, United States of America; 3 Institute for Human Infections and Immunity, UTMB, Galveston, Texas, United States of America; Universidade Federal de Minas Gerais, Brazil

## Abstract

Macrophage activation of NAD(P)H oxidase (NOX2) and reactive oxygen species (ROS) is suggested to kill *Trypanosoma cruzi* that causes Chagas disease. However, the role of NOX2 in generation of protective immunity and whether these mechanisms are deregulated in the event of NOX2 deficiency are not known, and examined in this study. Our data showed that C57BL/6 p47^phox−/−^ mice (lack NOX2 activity), as compared to wild-type (WT) mice, succumbed within 30 days post-infection (pi) to low doses of *T. cruzi* and exhibited inability to control tissue parasites. P47^phox−/−^ bone-marrow and splenic monocytes were not compromised in maturation, phagocytosis and parasite uptake capacity. The deficiency of NOX2 mediated ROS was compensated by higher level of inducible nitric oxide synthase (iNOS) expression, and nitric oxide and inflammatory cytokine (TNF-α, IFN-γ, IL-1β) release by p47^phox−/−^ macrophages as compared to that noted in WT controls infected by *T. cruzi*. Splenic activation of Th1 CD4^+^T cells and tissue infiltration of immune cells in *T. cruzi* infected p47^phox−/−^ mice were comparable to that noted in infected control mice. However, generation and activation of type 1 CD8^+^T cells was severely compromised in p47^phox−/−^ mice. In comparison, WT mice exhibited a robust *T. cruzi*-specific CD8^+^T cell response with type 1 (IFN-γ+TNF-α>IL-4+IL-10), cytolytic effector (CD8^+^CD107a^+^IFN-γ^+^) phenotype. We conclude that NOX2/ROS activity in macrophages signals the development of antigen-specific CD8^+^T cell response. In the event of NOX2 deficiency, a compromised CD8^+^T cell response is generated, leading to increased parasite burden, tissue pathogenesis and mortality in chagasic mice.

## Author Summary

Macrophage activation of NADPH oxidase NOX2) and reactive oxygen species (ROS) is suggested to mediate control of *Trypanosoma cruzi* infection that is the causative agent of Chagas disease. However, how NOX2/ROS deficiency affects parasite persistence and chronic disease is not known. In this study, we present the first evidence that NOX2 and ROS shape the T cell-mediated adaptive immunity, and its deficiency result in compromised splenic activation of type 1 cytotoxic CD8^+^ T cell response to *T. cruzi* infection. Subsequently, p47^phox−/−^ mice that lack NOX2 activity were more unable to control parasite replication and dissemination and succumbed to susceptible to *T. cruzi* infection. Our study highlights how redox state of innate immune cells alters the adaptive immunity to intracellular pathogens; and suggests that understanding the molecular and cellular mechanisms affected by redox state of immune cells at basal level could be exploited in designing future therapeutic and vaccination strategies against *T. cruzi* infection and Chagas disease.

## Introduction

Chagas disease is caused by the protozoan *Trypanosoma cruzi* [1], [2]. During acute phase of infection, parasites can be found in the circulating blood, and host may develop fever or swelling around the site of inoculation, and rarely, severe inflammation in heart muscle or brain. Several years after exposure to *T. cruzi*, ∼30% of the infected individuals develop clinical symptoms of chronic cardiomyopathy associated with progressive cardiomegaly, arrhythmia, thromboembolic events, and heart failure [3], [4].

Both innate and acquired immune responses are required for control of *T. cruzi* and critical for host survival (reviewed in [5], [6]). Upon infection, macrophages serve as first responders by activation of phagocytic NADPH oxidase, referred as NOX2. NADPH oxidase is a multi-subunit complex and utilizes NADPH as an electron donor to reduce O_2_ to superoxide (O_2_^·−^), that is then dismutated into other oxidants (e.g. H_2_O_2_) [7]. The plasma membrane-associated proteins gp91^phox^ and p22^phox^ compose the flavocytochrome-b558 complex that is the major component responsible for enzyme stability and activity. Phosphorylation of cytosolic factors (p47^phox^, p67^phox^, and p40^phox^), and small Rho GTPases in response to exogenous or endogenous stimuli initiates their translocation to the cell membrane, and NADPH oxidase activation [7]–[9]. Activated phagocytes exert cytotoxic effects via NOX2-dependent reactive oxygen species (ROS) production that mediates pathogen killing by oxidative damage of DNA, proteins and lipids, and suggested to play an important role in control of *T. cruzi* [10]–[14].

Besides innate immune mechanisms, a body of literature demonstrates that adaptive immune responses are required for parasite control. CD4^+^T cells assist in the control of *T. cruzi* through secretion of Th1 cytokines, amplification of the phagocytic activity of macrophages, stimulation of B cell proliferation and antibody production, and enhancement of the CD8^+^T cells response (reviewed in [6], [15]. CD8^+^T cells recognize processed parasite antigens presented in association with MHC class I molecules on the surface of infected host cells and contribute to the control of *T. cruzi*, either by cytolysis of parasite-infected cells or by the secretion of cytokines that may induce trypanocidal activity (reviewed in [6], [16]). Current literature suggests that NADPH oxidase activity may modulate adaptive immune responses via ROS signaling of cytokine gene expression and regulation of the efficient antigen presentation for T cell activation and proliferation [17], [18], though the cell type involved in NADPH oxidase-mediated regulation of adaptive immunity are not fully detailed.

In this study, we have assessed the host response to *T. cruzi* infection in the event of phagocytic NADPH oxidase deficiency. We first monitored the susceptibility of wild-type (WT) versus p47^phox−/−^ mice to *T. cruzi* infection, and then proceeded with a step-wise approach to identify the immune mechanisms that may be altered and contributed to susceptibility of p47^phox−/−^ mice to *T. cruzi*. Our data show that p47^phox−/−^ macrophages were not compromised in phagocytic activity, and mounted enhanced levels of inducible nitric oxide synthase (iNOS), nitric oxide (NO), and cytokines in response to *T. cruzi* infection. *In vivo* activation of CD4^+^T cell subset and inflammatory cytokine response was also similar to or more pronounced in p47^phox−/−^ mice when compared to that observed in WT controls in response to *T. cruzi* infection. However, in the event of NOX2 deficiency, generation and activation of CD8^+^T cell response was severely compromised leading to increased parasite burden, tissue pathogenesis and mortality. We discuss the involvement of distinct innate receptor signaling pathways governing the activation and proliferation of T cell subsets and the various mechanisms contributing to increased susceptibility of p47^phox−/−^ mice to *T. cruzi* infection.

## Results

### Susceptibility of p47^phox−/−^ mice to *T. cruzi* infection

**Figure 1:**
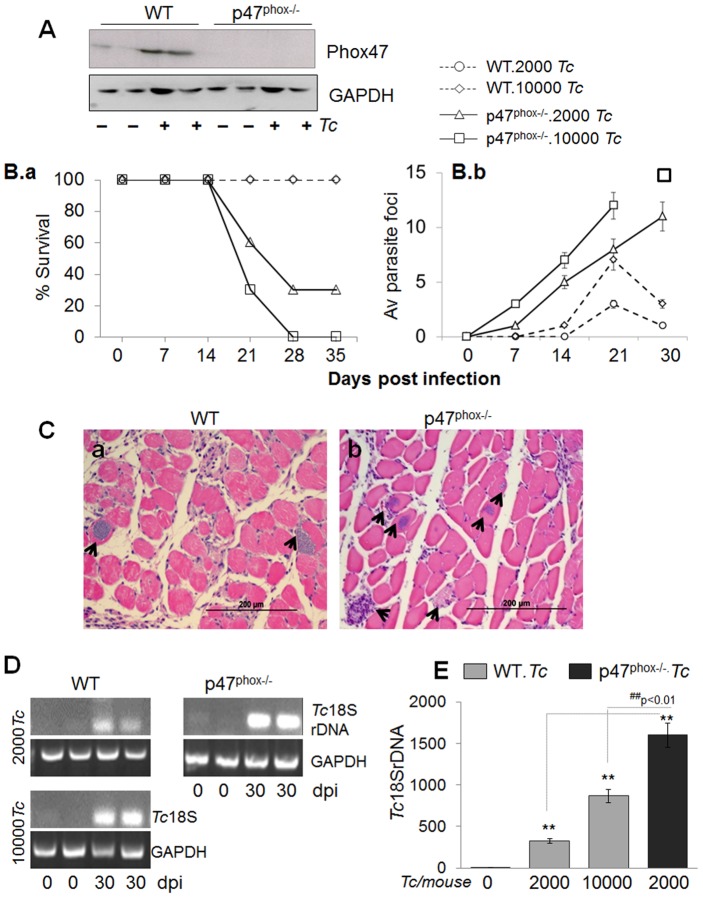
p47^phox−/−^ mice are susceptible to *T. cruzi* infection and exhibit increased mortality and parasite burden. (**A**) Western blotting of splenic macrophages and heart tissue, probed with anti-p47^phox^ antibody. The data presented are from mice infected with 10,000 parasites and harvested at day 7 pi. (**B–E**) C57BL/6 (WT and p47^phox−/−^) mice were infected with *T. cruzi* (2,000 or 10,000 parasites/mouse). (**B.a**) Percent survival from infection was monitored daily. (**B.b**) Tissue parasite foci in skeletal muscle tissue sections of infected mice were monitored by light microscopy at weekly intervals. (**C**) Histologic examination of heart tissue-sections for parasite foci (data shown is at day 30 post-infection, original magnification: 200×; arrows mark the parasite nests). (**D&E**) Semi-quantitative PCR (***D***) and real time quantitative PCR (***E***) analysis of parasite burden in the heart tissue of infected mice at day 30 pi. Data in all figures are presented as mean ± SD and significance presented as *normal-versus-infected, ^#^WT/infected-versus-p47^phox−/−^/infected (*^, #^ *p*<0.05, **^,##^ *p*<0.01, n = 8/group).

We used well-established experimental models [19], [20] to assess the role of NAD(P)H oxidase (NOX2) in immunity to *T. cruzi* infection. C57BL/6 (WT and p47^phox−/−^) mice were assessed at day 7 post-infection (pi) for the expression level of p47^phox^ as an indicator of NOX2 activation in innate immune cells. The low level of baseline expression of p47^phox^ was increased by 2-fold in splenic (Fig. 1A) and bone-marrow monocytes/macrophages of WT mice. The splenic and BM monocytes of p47^phox−/−^ mice exhibited no expression of p47 before or after *T. cruzi* infection. These data confirmed that p47^phox−/−^ mice lacked the ability to induce NOX2 activity in phagocytes in response to *T. cruzi* infection.

Challenge infection with 10,000 *T. cruzi* per mouse proved to be lethal for p47^phox−/−^ mice as all mice succumbed within 28 days pi (Fig. 1B.a). When inoculum was reduced to 2000 parasites, 70% of p47^phox−/−^ mice still succumbed by 30 days pi. In comparison, 100% of WT mice challenged with 2000 or 10000 parasites survived (Fig. 1B.a).

The increased mortality of p47^phox−/−^ mice was associated with increased tissue parasites (Fig. 1B.b, Fig. 1.C–E). Histological analysis of skeletal muscle and heart tissue sections (three sections/tissue >10-microscopic fields (mf) per slide, n = 8 mice/group) was conducted to obtain a score of parasite foci in tissues (Table 1). An average of skeletal tissue parasite foci in WT and p47^phox−/−^ mice infected with 2000 or 10000 parasites is presented in Fig. 1B.b. The p47^phox−/−^ mice infected with 2000 or 10000 parasites exhibited an early increase in tissue parasitemia by day 7 pi that further increased in a linear manner at days 14 and 21 pi (Fig. 1B.b). In comparison, WT mice exhibited a delayed, 2–5-fold lower level of parasite foci in skeletal muscle tissue during 7–21 days pi (Fig. 1B.b). At 30 days pi, parasite foci in WT mice infected with 2000 or 10000 parasites were controlled, while p47^phox−/−^ continued to exhibit an increase in tissue parasite foci (Fig. 1B.b). A similar pattern of increase in parasite foci was observed in heart tissue of p47^phox−/−^ mice during the 7–30 days pi (Table 1). We noted >5 parasite foci/mf in heart tissue sections of p47^phox−/−^ mice infected with 2000 or 10000 parasites and harvested at day 30 pi (Fig. 1C.b). In comparison, contained (0–2 pseudocysts/mf) were noted in heart tissue of WT mice infected with 2000 or 10000 parasites (Fig. 1C.a, Table 1) A semi-quantitative PCR showed the *Tc18SrDNA* signal was significantly higher in the myocardium of infected/p47^phox−/−^ mice at day 30 pi than was observed in the myocardium of WT mice infected with the same dose or 5-fold higher dose of parasites (Fig. 1D). Quantitative real-time PCR validated the findings of semi-quantitative PCR and showed 2–5-fold increase in myocardial (Fig. 1E, ^##^p<0.01), and skeletal muscle and circulatory parasite burden in infected/p47^phox−/−^ mice as compared to that detected in infected/WT mice. These data suggested the p47^phox−/−^ mice failed to control tissue parasites and succumbed to *T. cruzi* infection.

**Table 1:** Tissue burden of parasite foci and inflammation in wild type and p47^phox−/−^ mice infected by T. cruzi.

	Wild type				p47^phox−/−^			
Days post-infection	10,000 *T. cruzi*		2,000 *T. cruzi*		10,000 *T. cruzi*		2,000 *T. cruzi*	
	Sk mus	Heart	Sk mus	Heart	Sk mus	Heart	Sk mus	Heart
	**Parasitic pseudocysts/mf**							
0	0	0	0	0	0	0	0	0
7	0	0	0	0	2	0	0	0
14	1	0	0	0	7	0–3	5	0–3
21	7	0–1	3	0–1	12	0–4	8	0–3
30	3	0–1	1	0–1	ND	1–5	11	1–5
	**Myocarditis/inflammation score/mf**							
0	0	0	0	0	0	0	0	0
7	0	0	0	0	0–2	0–1	0–1	0–1
14	2	1	0–1	0	3–4	1–2	2	0–2
21	2–3	1–2	1–2	0–1	3–4	3	3	2
30	3	1–2	2	0–1	3–4	ND	4	2

C57BL/6 mice (wild type and p47phox−/−) were infected with T. cruzi, as detailed in Materials and Methods. Tissue sections (skeletal muscle, heart) were stained with H&E. In general, we analyzed each tissue-section for >10-microscopic fields (100× magnification), and examined three different skeletal muscle or left ventricular (LV) tissue sections/mouse (4 mice/group) to obtain a semi-quantitative score of parasitic foci (cells filled with parasites). Myocarditis (presence of inflammatory cells) was scored as 0 (absent), 1 (focal or mild with ≤1 foci), 2 (moderate with ≥2 inflammatory foci), 3 (extensive with generalized coalescing of inflammatory foci or disseminated inflammation), and 4 (diffused inflammation with severe tissue necrosis, interstitial edema, and loss of integrity).

### Phagocytic activity of P47^phox−/−^ macrophages is not compromised

One plausible explanation for increased susceptibility of p47^phox−/−^ mice to *T. cruzi* could be that p47^phox−/−^ macrophages were compromised in phagocytic activity, and, therefore, failed to control parasites' dissemination. To test this, we isolated BM and splenic monocytes from WT and p47^phox−/−^ mice, *in vitro* differentiated to macrophages with interferon gamma (IFN-γ), and incubated in presence of *T. cruzi* for 0, 6, 12, 24 h. The data presented in Fig. S1 are from splenic monocytes and representative of the results from triplicate experiments with splenic and BM monocytes. Giemsa staining showed the monocytes of p47^phox−/−^ mice had a similar or better capacity than the WT monocytes to differentiate to macrophages by 6 h pi (Fig. S1.b&g). Likewise, p47^phox−/−^ phagocytes' capacity to uptake parasites (i.e. phagocytic efficiency) was not significantly compromised. Counting of >200 cells/slide showed that by 6 h, 25% and 15% of WT and p47^phox−/−^ macrophages were infected (average 6–15 parasites/cell), and at 12 h, >50% of WT and p47^phox−/−^ macrophages were full of replicative, amastigote form of parasites (Fig. S1.c,d,h,i). At 24 h pi, some of the WT macrophages were ruptured releasing parasites while p47^phox−/−^ macrophages continued to exhibit parasites contained within phagosome (Fig. S1.e&j, p<0.01).

**Figure 2:**
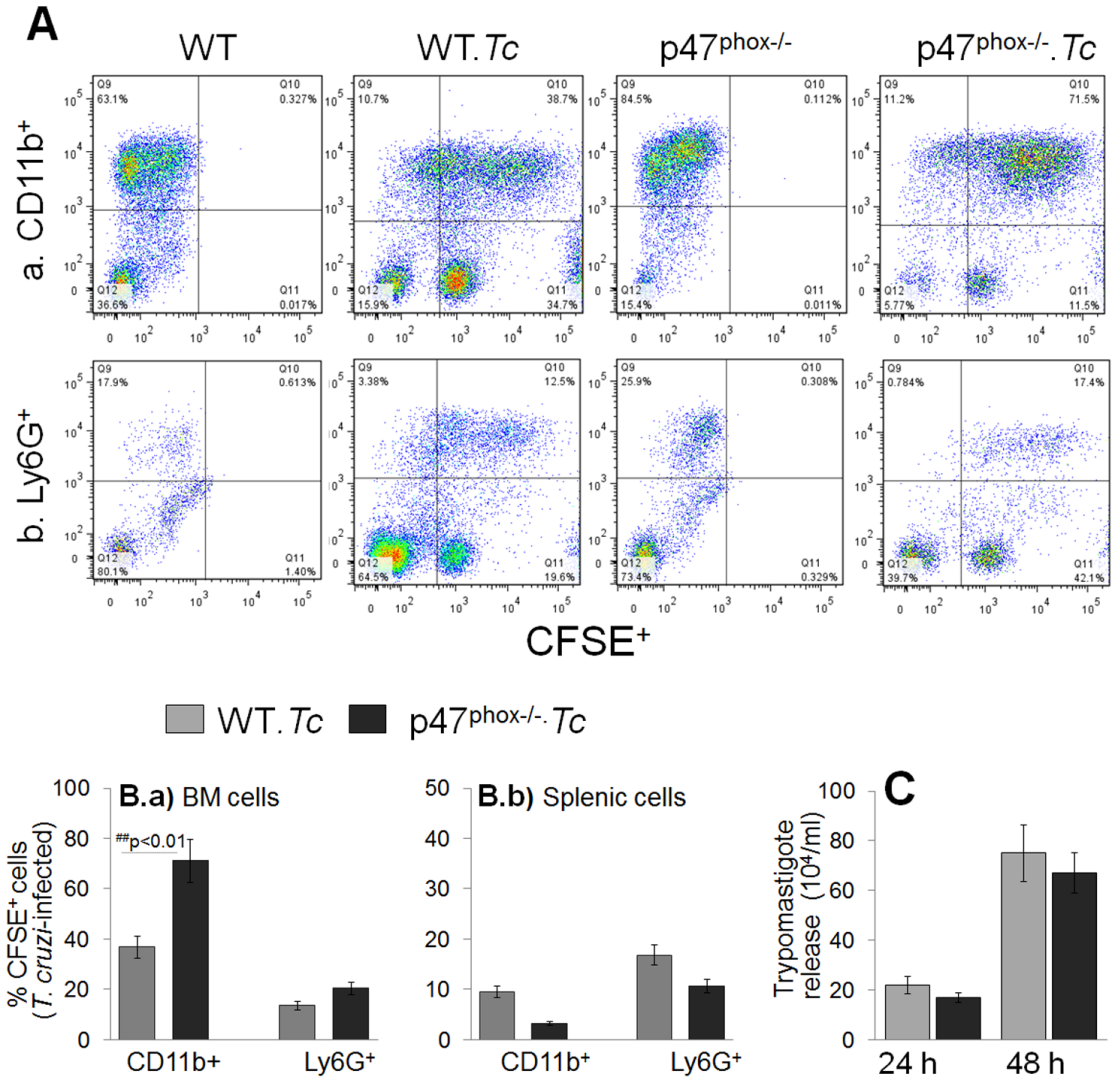
*T. cruzi* uptake, intracellular replication and release by p47^phox−/−^ phagocytes. (**A**) Bone marrow (BM) and splenic cells were isolated from WT and p47^phox−/−^ mice and *in vitro* incubated for 24 h with CFSE-labeled *T. cruzi* followed by 30 min with fluorescence conjugated anti-CD11b (macrophage marker) and anti-Ly6G (neutrophil marker) antibodies. The antibody-labeled cells were fixed, and analyzed by flow cytometry. Representative flow cytometry analyses of BM cells infected with CFSE-labeled *T. cruzi* are shown. (**B**) Bar graphs of the percentage of BM (***Ba***) and splenic (***Bb***) cell populations (CFSE^+^CD11b^+^: infected macrophages, CFSE^+^Ly6^+^: infected neutrophils). Data are derived from 3 independent experiments. (**C**) Supernatants of infected splenic monocytes were monitored for parasite release at 24 h and 48 h post-incubation by light microscopy. Data are presented as mean ± SD and significance presented as *normal-versus-infected, ^#^WT/infected-versus-p47^phox−/−^/infected (*^, #^ *p*<0.05, **^,##^ *p*<0.01.

To obtain a quantitative measure of parasite uptake, primary BM and splenic cells were incubated for 24 h with CFSE-labeled *T. cruzi*, and then labeled with fluorescence-conjugated antibodies to examine the frequency of CFSE^+^ macrophages (APC-CD11b^+^) and neutrophils (PE-Ly6B^+^) by flow cytometry. Representative flow cytometry data from BM-macrophages and BM-neutrophils incubated with CFSE-labeled parasites are presented in Fig. 2A, and percentage of CFSE^+^CD11b^+^ and CFSE^+^Ly6B^+^ macrophages and neutrophils, respectively, from BM and splenic cells are presented in Fig. 2B. We noted a higher extent of infection of BM cells derived from p47^phox−/−^ mice as compared to that noted in BM cells from WT mice (CD11b^+^ macrophages: 71% versus 36%; Ly6^+^ neutrophils: 23.5% versus 13.5%; p47^phox−/−^ versus WT, respectively, Fig. 2A & Fig. 2B.a). The splenic cells from p47^−/−^ and WT mice exhibited comparable rate of infection efficiency at 24 h post-incubation that were not statistically different (CD11b^+^ macrophages: 10.65% versus 16.8%; Ly6+ neutrophils: 3.28% versus 9.45%, p47^phox−/−^ versus WT, respectively, Fig. 2B.b). The hemacytometer counting of parasites in supernatants showed comparable number of parasites were released from p47^phox−/−^ and WT macrophages at 24 h and 48 h pi (Fig. 2C). Together, the data presented in Fig. S1 and Fig. 2 suggested that BM and splenic monocytes from WT and p47^phox−/−^ mice were equally competent in differentiating to macrophages and parasite uptake, and NOX2 deficiency did not result in increased parasite release from infected macrophages.

### *In vitro* functional activation of p47^phox−/−^ macrophages in response to *T. cruzi*

**Figure 3:**
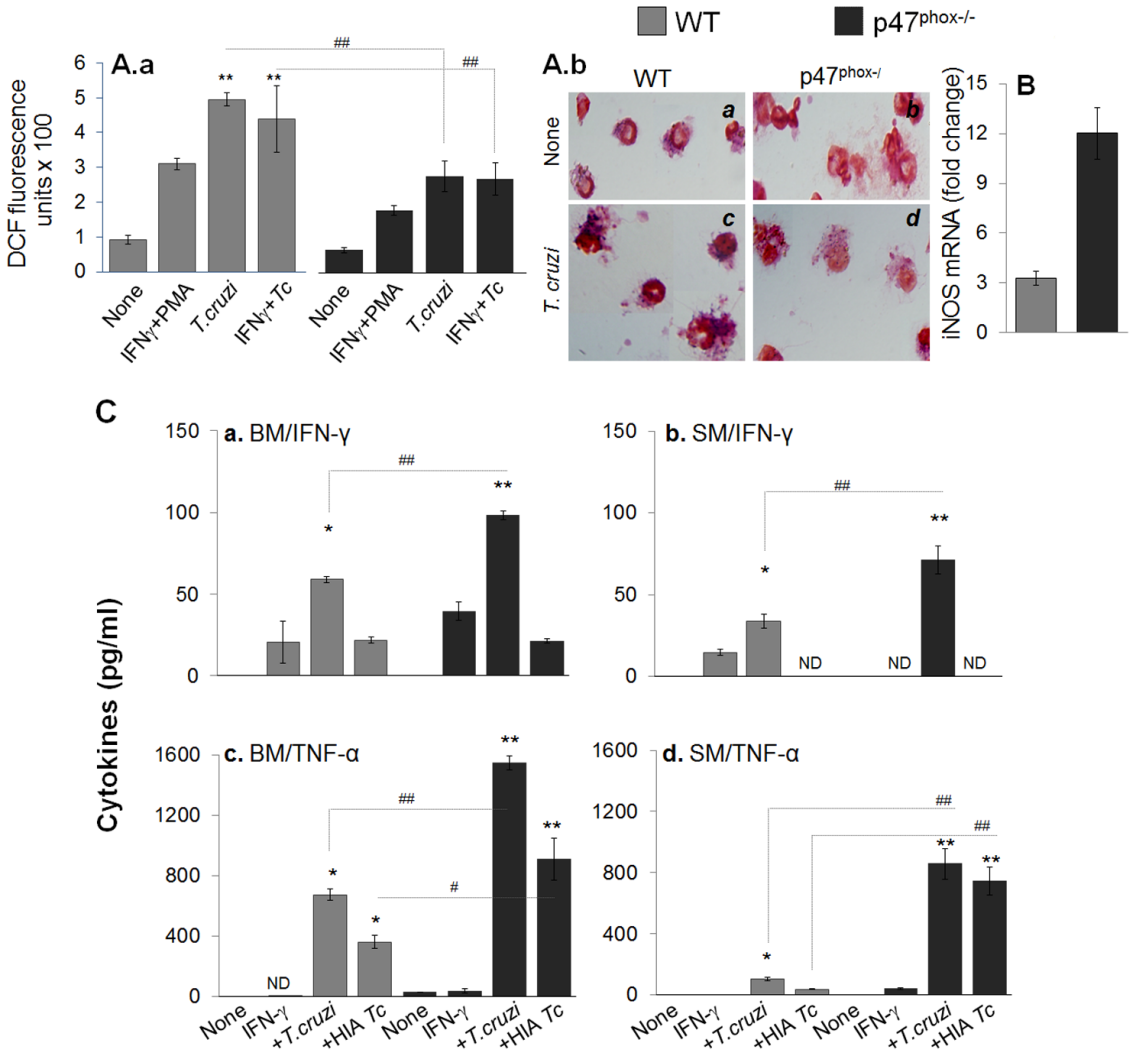
*In vitro* functional activation of p47^phox−/−^ macrophages in response to *T. cruzi* infection. (**A&B**) Isolated splenocytes from WT and p47^phox−/−^ mice were *in vitro* infected with live or heat-inactivated (HIA) *T. cruzi* for 24 h (± IFNγ). Cells incubated with phorbol myristate acetate (PMA) were used as positive controls. (**A.a**) H_2_DCFDA oxidation by intracellular ROS was monitored by fluorimetry. (**A.b**) Nitroblue tetrazolium (NBT) reduction to formazan blue crystals by cellular superoxide was visualized by light microscopy. (**B**) RT-PCR quantitation of iNOS mRNA level. (**C**) BM (panels a&c) and splenic (panels c&d) cells were stimulated with IFN-γ, washed, and then exposed to *T. cruzi* (live or heat-inactivated) for 24 h. The release of IFN-γ (panels a&b) and TNF-α (panels c&d) in supernatants was monitored by an ELISA. ND: not detectable.

Macrophages control the invading pathogen through production of ROS, NO, and inflammatory cytokines. Isolated BM and splenic monocytes from WT and p47^phox−/−^ mice were incubated for 0, 6, 12 and 24 h with *T. cruzi* (± recombinant IFN-γ). We measured ROS levels using H_2_DCFDA that is cell permeable and when oxidized by ROS, releases fluorescent DCF. A gradual increase in DCF fluorescence beginning at 6 h that reached the maximal level at 24 h pi was observed in WT macrophages. Macrophages from WT mice responded to *T. cruzi* infection (± rIFN-γ) by 5-fold increase in DCF fluorescence (Fig. 3A.a). The p47^phox−/−^ splenic macrophages (±rIFN-γ) exhibited 2.5-fold lesser DCF fluorescence in response to *T. cruzi* when compared to that noted in infected/WT cells (Fig. 3A.a, ^##^p<0.01). Likewise, the p47^phox−/−^ BM macrophages (± rIFN-γ) exhibited a 2-fold decline in ROS levels as compared to that noted in WT BM macrophages upon *T. cruzi* infection. The superoxide-dependent formation of formazan blue crystals was noted to be significantly increased in WT and slightly increased in p47^phox−/−^ splenic macrophages incubated for 24 h with *T. cruzi* (Fig. 3A.b). *T. cruzi*-induced increase in DCF fluorescence and nitroblue tetrazolium (NBT) reduction was quenched by >90% when cells were incubated in presence of 0.5 mM apocynin (inhibits NADPH oxidase activity) or 10 µM N-acetyl cysteine (ROS scavenger), suggesting the observed increase in ROS is primarily due to NOX-dependent ROS from infected macrophages. The iNOS mRNA level, determined by qRT-PCR, was increased by 4-fold in p47^phox−/−^ splenocytes as compared to that noted in WT controls, infected *in vitro* for 24 h (Fig. 3B). The release of cytokines in supernatants of primary BM and splenic cells incubated with *T. cruzi* for 24 h was measured by an ELISA. The p47^phox−/−^ BM and splenic cells responded to *T. cruzi* by >10-fold increase in IFN-γ and TNF-α release that was significantly higher than that observed in infected/WT cells (Fig. 3C). Together the data presented in Fig. 3 suggested that p47^phox−/−^ macrophages lacked the ability to mount a strong NOX2-dependent ROS; however, exhibited a higher extent of iNOS and proinflammatory cytokine expression in response to *T. cruzi* infection. The p47^phox−/−^ monocytes/macrophages also responded to heat-inactivated *T. cruzi* with a strong proinflammatory cytokines production.

### Tissue inflammatory infiltrate in p47^phox−/−^ mice infected by *T. cruzi*

**Figure 4:**
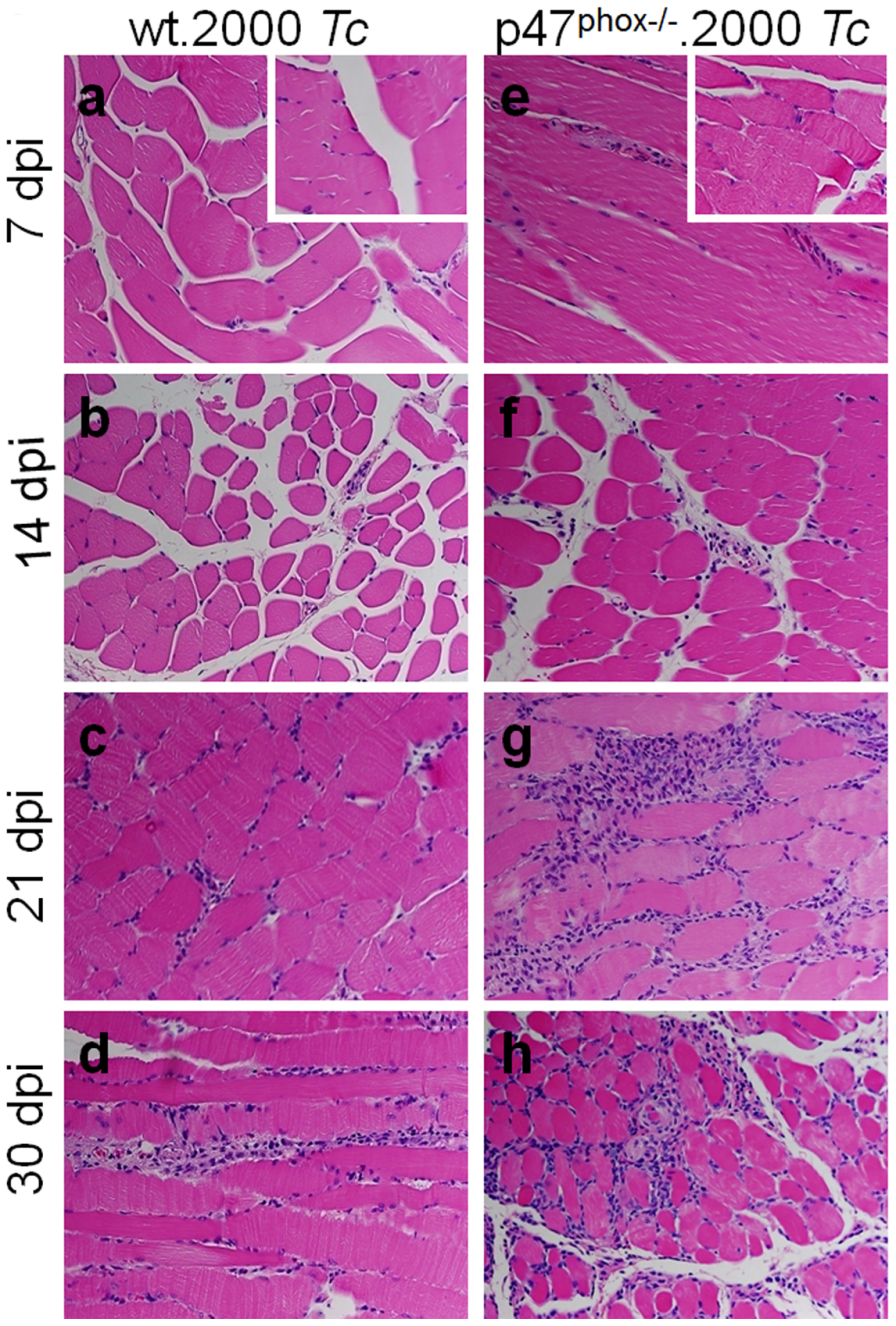
Histological analysis of inflammatory infiltrate in *T. cruzi*-infected p47^phox−/−^ mice. Shown are representative images of H&E staining (blue: nuclear, pink: muscle/cytoplasm/keratin) of skeletal muscle sections from WT (panels a–d) and p47^phox−/−^ (panels e–h) mice infected with *T. cruzi* (2000 parasites/mouse), and harvested at day 7 (a&e), 14 (b&f), 21 (c&g), and 30 (d&h) post-infection. H&E stained images from normal mice are shown as insets in panels a and e (magnification: 40×).

To further gain an indication of the effects of phagocytes' NOX2 deficiency on host immunity to *T. cruzi*; we looked at the tissue infiltration of immune cells in *T. cruzi* infected WT and p47^phox−/−^ mice by histological studies (Fig. 4, Fig. S2, and Table 1). The p47^phox−/−^ mice injected with 2000 parasites exhibited infiltration of inflammatory infiltrate in skeletal muscle and heart tissue as early as day 7 post-infection (pi, Fig. 4.e, Table 1). The inflammatory foci were observed in all tissue sections by 14 days pi (score: 2), and extensive inflammation with large inflammatory foci or diffused inflammation throughout the tissue section (score: 2–4) was observed at 21–30 days pi in skeletal muscle (Fig. 4.f–h) and heart tissue (Fig. S1.m–o) of p47^phox−/−^ mice. In WT mice, infection with 2000 parasites resulted in minimal inflammation of the skeletal muscle (Fig. 4.a&b) and heart tissue (Table 1) at 7–14 days pi; and inflammatory infiltrate was moderately increased (score: 1–2) at 21–30 days pi (Fig. 4.c&d, Table 1). These data suggested that p47^phox−/−^ mice responded to *T. cruzi* infection (2000 parasites/mouse) with an increase in tissue infiltration of inflammatory infiltrate that was higher than that observed in WT mice given the same dose of parasites. Infection with a 5-fold higher dose of parasites was required to elicit the extent of increase in inflammatory infiltrate in skeletal muscle (score: 2–4, Fig. S1.d&e) and heart tissue (score: 1–2, Fig. S1.i&j) of WT mice as was noted in skeletal muscle and heart tissue of p47^phox−/−^ mice infected with 2,000 parasites.

### Functional response of splenocytes and BM cells in p47^phox−/−^ mice infected with *T. cruzi*

**Figure 5:**
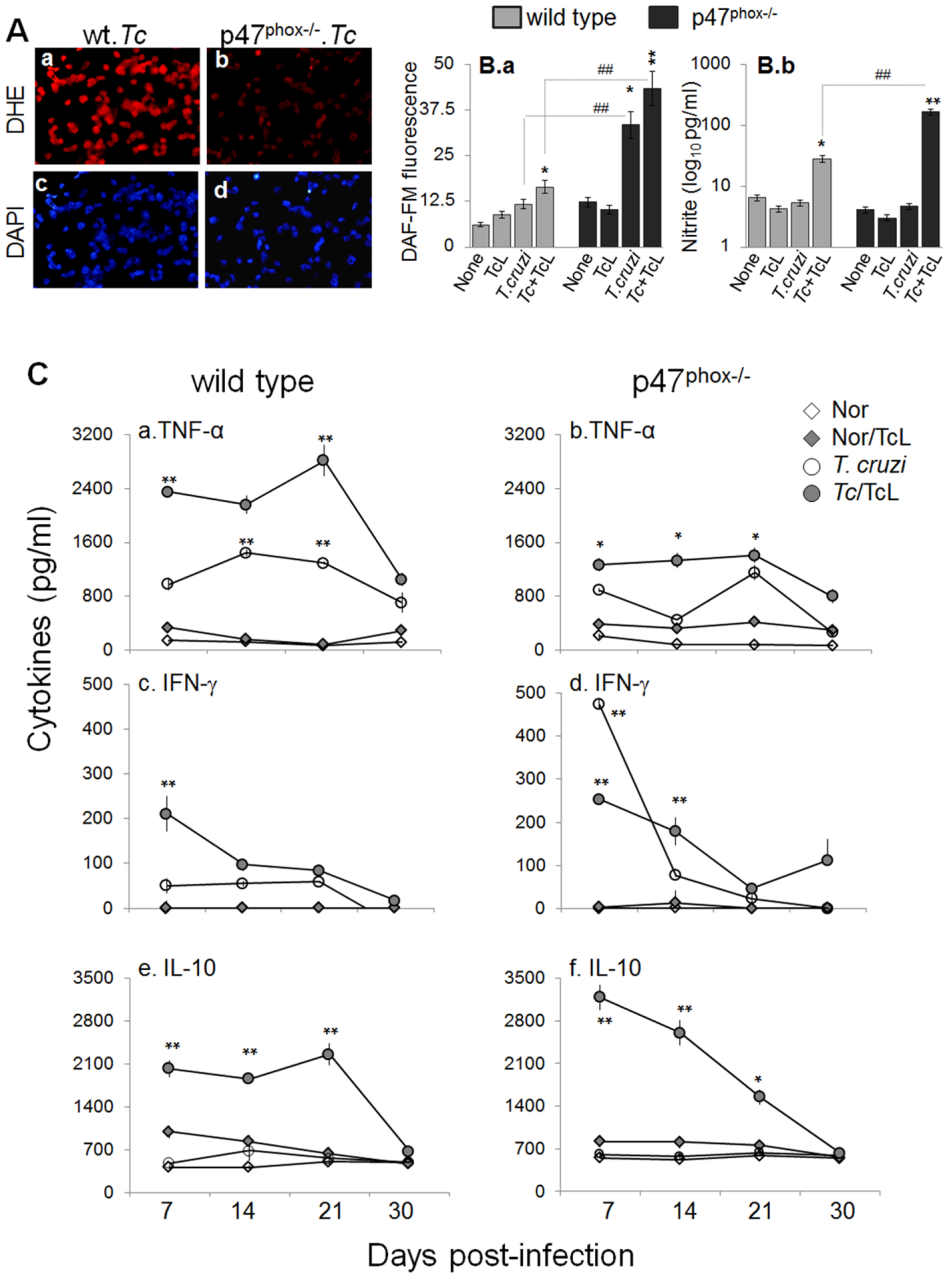
*In vivo* ROS, nitric oxide, and cytokine profile in p47^phox−/−^ mice infected with *T. cruzi*. Mice (WT and p47^phox−/−^) were infected with *T. cruzi* as described in Materials and Methods. (**A**) Splenic single cell population from infected mice (30 dpi) were cyto-spinned on glass slides. Shown is fluorescence staining for dihydroethidium (measures intracellular ROS, binds DNA). Cells stained with DAPI (binds nuclei, blue) are shown as controls. (**B**) Splenocytes were harvested from *T. cruzi*-infected mice at day 30 pi, and *in vitro* incubated for 24 h (± TcL). Shown is intracellular nitric oxide (panel a) and nitrite levels in the supernatant (panel b), determined by using DAF-FM staining/fluorimetry and Griess reagent assay, respectively. (**C**) Splenocytes were harvested from infected mice at day 7, 14, 21, and 30 post-infection, and incubated *in vitro* (± TcL) for 48 h. Shown are the TNF-α (a&b), IFN-γ (c&d) and IL-10 (e&f) levels in cell-free supernatants, determined by an ELISA. ND: not detectable.

To examine the quality of inflammatory response *in vivo*, WT and p47^phox−/−^ mice were harvested at day 7, 14, 21, and 30 post-infection. BM and splenic cells from infected mice were either directly analyzed or *in vitro* stimulated in presence of *T. cruzi* trypomastigote lysate (TcL) and utilized for functional assessment. Shown in Fig. 5A are intracellular ROS levels in splenic cells of infected mice at day 30 pi, determined by dihydroethidium (DHE) fluorescence. DHE is cell permeable, and when oxidized to ethidium, accumulates in nuclei and fluoresces bright red. We noted a significant increase in ethidium fluorescence in splenocytes of infected/WT, but not of infected/p47^phox−/−^ mice, at all time-points pi (Fig. 5A.a&b). Likewise, BM cells isolated at day 7, 14, 21, and 30 from infected/WT mice, but not from infected/p47^phox−/−^ mice, exhibited a significant increase in DHE fluorescence. DHE fluorescence was quenched when cells were incubated in presence of 0.5 mM apocynin (NOX2 inhibitor). Note that 4′-6-diamidino-2-phenylindole-dihydrochloride (DAPI, binds nuclear DNA) staining of the splenocytes (Fig. 5A.c&d) of infected/WT and infected/p47^phox−/−^ mice was comparable.

The intracellular nitric oxide levels in BM and splenic cells harvested at day 7, 14, 21 and 30 pi was first determined by DAF-FM-based fluorimetry. DAF-FM is cell permeable, and forms fluorescent benzotriazole upon reaction with nitric oxide. Shown in Fig. 5B.a are arbitrary units of DAF-FM fluorescence in splenic cells of infected mice harvested at day 30 pi. Our data showed a 4-fold increase in DAF-FM fluorescence in splenocytes of infected/p47phox−/− mice that was further increased upon *in vitro* stimulation with TcL. In comparison, splenic cells of infected/WT mice exhibited a significant increase in intracellular nitric oxide (DAF-FM fluorescence) only after secondary *in vitro* stimulation with TcL, and this response was ∼3-fold lesser than that observed with splenocytes of infected/p47phox−/− mice (Fig. 5B.a). Because DAF-FM may exhibit non-specific signal by reacting with N compounds others than nitric oxide, we also performed a Griess reagent assay to evaluate the nitric oxide production rate, reflected by nitrite release. Splenocytes of infected/p47^phox−/−^ mice, *in vitro* stimulated with TcL, exhibited a robust increase in nitrite release that was >5.8-fold higher than that noted with splenic cells from infected/WT mice (Fig. 5B.b). Likewise, BM monocytes of infected/p47^phox−/−^ mice responded to *in vitro* antigenic stimulus (TcL) by a robust 7-fold and 8-fold increase in DAF-FM fluorescence and nitrite release, respectively. The extent of TcL-stimulated nitrite release was 4-fold (32.8±4.7 versus 8.07±0.6 pg nitrite/ml) higher in BM cells of infected/p47^phox−/−^ mice than that noted in BM cells of infected/WT mice (^##^p<0.001). In all experiments, incubation of splenic or BM monocytes from infected mice with 5 µmol/ml L-NAME (inhibits iNOS activity/nitric oxide) abolished the DAF-FM fluorescence and nitrite release.

To examine the *in vivo* cytokine profile in response to infection, BM and splenic cells from WT and p47^phox−/−^ mice were harvested at 7, 14, 21, and 30 days pi, *in vitro* incubated with or without second antigenic stimulus for 48 h, and supernatants were submitted to an ELISA. Overall, splenocytes of WT and p47^phox−/−^ mice (± *T. cruzi* lysate) were activated early upon infection, as is evidenced by a significant increase in TNF-α, IFN-γ and IL-10 levels at day 7 pi (Fig. 5C). No IL-4 release was observed. The splenocytes of infected/WT mice exhibited a predominance of TNF-α (TNF-α>IL-10>IFN-γ) release throughout the course of infection (Fig. 5C.a,c,e). In comparison, splenocytes of infected/p47^phox−/−^ mice (± TcL) exhibited a mixed response with a predominance of IL-10 (IL-10>TNF-α>IFN-γ) at 7, 14, 21 and 30 days post-infection (Fig. 5C.b,d,f). The BM cells of WT and p47^phox−/−^ mice infected with *T. cruzi* (±TcL) exhibited a similar pattern of cytokine response as was noted in splenocytes. Together, the data presented in Fig. 5 suggested that compromised ROS production capacity due to NOX2 deficiency was compensated by an increased iNOS and nitric oxide levels in p47^phox−/−^ mice infected with *T. cruzi*. However, p47^phox−/−^ mice exhibited a subdued proinflammatory cytokine response (IL-10>TNF-α) during *T. cruzi* infection.

### Characterization of T cell response in p47^phox−/−^ mice infected by *T. cruzi*

**Figure 6:**
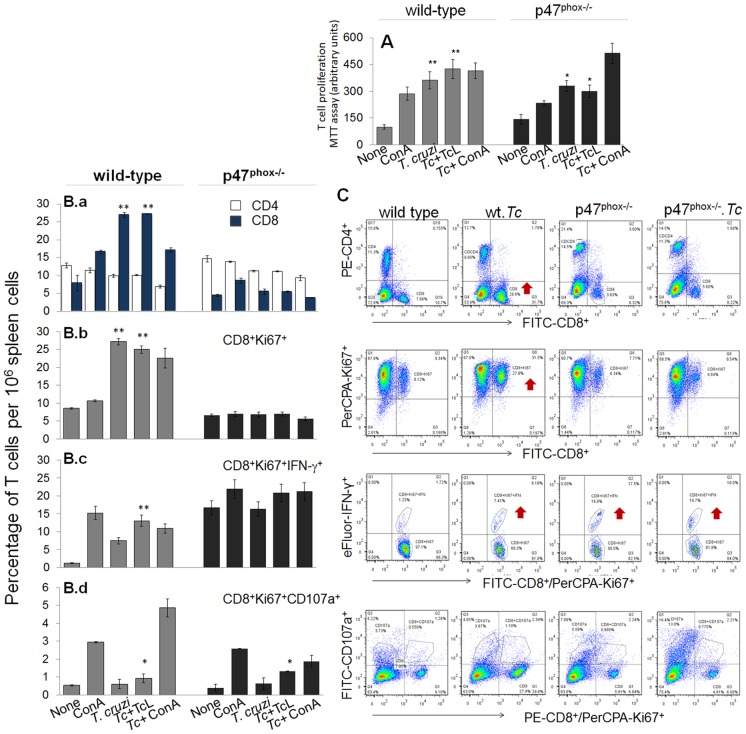
The p47^phox−/−^ mice lacked the ability to develop CD8^+^T cell response to *T. cruzi* infection. Mice were infected with *T. cruzi*, and harvested at day 30 pi. Splenocytes were *in vitro* incubated for 48 h in presence or absence of TcL as 2^nd^ antigenic stimulus (controls: concanavalin A). (**A**) Cell proliferation determined by an MTT assay. (**B&C**) After *in vitro* stimulation (±TcL), splenocytes from infected mice were labeled for 30-min with fluorescent- conjugated specific antibodies as described in Materials and Methods, and analyzed by flow cytometry. Splenic frequency of CD4^+^ and CD8^+^ T cells (B.a) are shown. Shown are also the mean percentage of CD8^+^ T cells that were Ki67^+^ (PerCPCy 5.5, B.b), IFN-γ^+^ (e-Fluor, B.c), and CD107a^+^ (PerCPA, B.d). Representative quandrant images of flow cytometry analysis of T cell subsets are shown in (**C**).

CD4^+^ and CD8^+^ T cells are important constituents of the adaptive immunity against *T. cruzi*. To gain an appreciation for the role of NOX2 in determining T cell functional profile, we evaluated the *in vivo* quality and potency of the cellular immune responses elicited in WT versus p47^phox−/−^ mice. Splenocytes, harvested at 30 days post-infection, were incubated in presence and absence of TcL antigenic stimulus, and T cell proliferation determined by an MTT assay (Fig. 6A). The CD4^+^ and CD8^+^ T cells were examined for proliferative capacity (Ki67^+^), intracellular cytokine profile (IFN-γ, TNF-α) and marker of lytic capacity (CD107a) by flow cytometry. The mean fluorescence intensity (±SD) indicative of T cell profile (Fig. 6B, n = 6/group) were derived from representative quadrant images of flow cytometry results presented in Fig. 6C. Splenic lymphocytes of WT mice exhibited 3.6–4.2-fold increase in proliferation in response to *T. cruzi* infection (± *in vitro* stimulation with TcL, Fig. 6A). In comparison, p47^phox−/−^ mice exhibited a ∼40% lower rate of splenic lymphocyte proliferation in response to *T. cruzi* infection, and no effect of *in vitro* stimulation with TcL was noted (Fig. 6A, ^##^p<0.01).

When we performed the specific T cell population analysis, we found that *in vivo* population of CD4^+^T cells was comparable in naïve WT and p47^phox−/−^ mice (range: 12–14%, Fig. 6B.a), and exhibited no significant change in proliferation (Ki67^+^) or IFN-γ^+^ phenotype in response to *T. cruzi* infection or subsequent incubation with TcL antigenic lysate. Instead, *in vivo* percentage of CD8^+^T cells in p47^phox−/−^ naïve mice (4.5%) was ∼2-fold lower than that noted in WT normal controls (7.9%) (Fig. 6B.a). In response to *T. cruzi* infection, CD8^+^T cells expanded by 3.4-fold in WT mice (27% of total) while these cells expanded at a very low frequency in p47^phox−/−^ mice (Fig. 6B.a). Functional characterization of CD8^+^T cells showed that a majority of the CD8^+^T cells were proliferative (Ki67^+^) in infected/WT mice (Fig. 6B.b) and a significant proportion of the CD8^+^Ki67^+^ cells (up to 13%) produced IFN-γ in an antigen-specific manner (Fig. 6B.c). In p47^phox−/−^ mice, CD8^+^T cells exhibited no significant proliferating phenotype in response to *T. cruzi* infection (Fig. 6B.b). Further, up to 16% of the CD8^+^T cells exhibited IFN-γ^+^ phenotype in naïve p47^phox−/−^ mice and these didn't increase in response to *T. cruzi* infection or second antigenic stimulation with TcL (Fig. 6B.b).

Transport of CD107a and CD107b to the plasma membrane of effector T cells is required for a) the cytolytic activity mediated by perforin and granzymes and b) the release of IFN-γ which exerts pleiotropic effects to suppress intracellular pathogens. Our data showed *T. cruzi* infection induced CD107a^+^CD8^+^T cells (2–4%) in infected/p47^phox−/−^ mice (Fig. 6B.d), and a majority of these exhibited dual-positive (CD107a^+^IFN-γ^+^) cytolytic phenotype, comparable to that noted in infected/WT mice. Together, the data presented in Fig. 6 suggested that splenic CD8^+^T cells in p47^phox−/−^ mice were low in number and failed to expand in response to *T. cruzi* infection, resulting in a substantial decline in proliferating, IFN-γ-producing cytolytic CD8^+^T cell response. In comparison, an expansive CD8^+^T cells proliferation that were predominantly IFN-γ^+^ with cytolytic capacity, and, thus, had a potential to act as effector T cells was induced in WT mice infected by *T. cruzi*.

## Discussion

The present study shows that in the absence of NOX2 activity, a defective activation of CD8^+^T cell occurs, and contributes to the inability of mice to successfully control *T. cruzi* infection. Our data suggested that the NOX2 deficiency was compensated by enhanced levels of iNOS, nitric oxide, and inflammatory cytokines in macrophages; however, p47^phox−/−^ mice were highly susceptible to *T. cruzi* because of the inability to activate a type 1 CD8^+^T cell response that is known to be essential for intracellular parasite control. Our study highlights how redox state of innate immune cells alters the adaptive immunity to intracellular pathogens, and understanding the molecular and cellular mechanisms affected by redox state of immune cells at basal level could be exploited in designing future vaccination strategies against *T. cruzi* infection and Chagas disease.

Current literature demonstrates that macrophage-derived free radicals (O_2_•^−^, nitric oxide) generated by the NOX2 complex and iNOS participate in cytotoxic mechanisms against microorganisms (reviewed in [21] [22]). In the context of *T. cruzi*, it is suggested that nitric oxide plays a central role through its action on macrophage-derived peroxynitrite formation, a strong cytotoxic oxidant that is formed by the reaction of nitric oxide with O_2_•^−^ [14], [23], [24]. Our *in vitro* studies showed that p47^phox−/−^ monocytes were better than (or equal to) the WT controls in their ability to differentiate into macrophages and phagocytize parasites (Fig. S1). Though it appeared that a higher number of intracellular parasites were present in infected p47^phox−/−^ macrophages at 24 h pi (Fig. S1 & Fig. 2); however, the extent of parasite release from p47^phox−/−^ cells at 24 and 48 h pi was comparable to that noted in WT controls (Fig. 2C), thus suggesting that NOX2 deficiency did not result in increased parasite survival in infected macrophages. Our observations are supported by others demonstrating the enhanced replication of bacteria (e.g. *Coxiella burnetii*) in p47^phox−/−^ macrophages, that was followed by a slightly delayed control of infection at a rate similar to the WT macrophages [25]. We propose that p47^phox−/−^ macrophages, despite a lack of NOX2/ROS, were equipped to phagocytize and control the parasites through compensatory mechanisms. One, a low but detectable level of O_2_•^−^ production in p47^phox−/−^ macrophages (Fig. 3A&B) was sufficient to support the nitric oxide mediated cytotoxic peroxynitrite formation for parasite killing. Indeed, O_2_•^−^ and nitric oxide can rapidly diffuse (diffusion control rates: 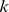
∼1010 m−1 s−1) and react to form peroxynitrite that is significantly more potent cytotoxin against trypomastigotes than H_2_O_2_ only [13], [14]. Secondly, a significant up regulation of the iNOS, nitric oxide and inflammatory cytokines (IFN-γ/TNF-α) in p47^phox−/−^ macrophages in response to *T. cruzi* infection (Fig. 3) could have controlled the infectious pathogen. Others have also demonstrated increased iNOS and nitric oxide levels in gp91^phox−/−^ mice infected by *T. cruzi* [26]. We surmise that in the event of defects in mounting NOX2/ROS, macrophages are capable of using alternative, compensatory mechanisms for pathogen control. Further studies will be required to conclusively establish if the peroxynitrite formation rate is indeed enhanced and identify the signaling mechanisms that were up regulated resulting in enhanced iNOS and inflammatory cytokines' expression in p47^phox−/−^ macrophages in response to *T. cruzi* infection.

The production of cytokines (IL-12, TNF-α) by innate immune cells (macrophages, dendritic cells (DCs) shapes the adaptive immunity via activation of T cells. CD4^+^ and CD8^+^ T cells producing type 1 cytokines and CD8^+^T cell mediated cytolytic activity are required for control of *T. cruzi* infection (reviewed in [6], [16], [27]. Our observation of increased release of IFN-γ/TNF-α by p47^phox−/−^ macrophages *in vitro* infected with *T. cruzi* (Fig. 3) suggest that NOX2/ROS might control the cytokinopathy via regulating the cytokine gene expression; however, NOX2 deficiency did not inhibit the phagocytes ability to provide inflammatory cytokine milieu for the recruitment and activation of T cells. Indeed flow cytometry analysis showed the CD4^+^T cells in p47^phox−/−^ mice responded to *T. cruzi* infection and/or *in vitro* antigenic stimulus by activation and proliferation to a similar extent as was noted in WT mice (Fig. 6). Others have shown that IFN-γ/LPS-treated p47^phox−/−^ mice secrete more IL-12 from DCs than similarly treated WT mice, and IFNγ/LPS matured p47^phox−/−^ DCs biased more ovalbumin-specific CD4^+^T cells toward a Th1 phenotype than the WT controls in a ROS-dependent manner [28]. It is also suggested that CD4^+^ T cells from p47^phox^ deficient mice exhibit augmented IFN-γ and diminished IL-4 production and an increased ratio of expression of T-bet (Th1-specific transcription factor) versus GATA-3 (Th2-specfic transcription factor), consistent with a Th1 skewing of naïve T cells [29]. Selective inhibition of TCR-induced STAT5 phosphorylation was identified as a potential mechanism for skewed helper CD4^+^T cell differentiation in p47^phox−/−^ mice [29]. We surmise that p47^phox^-dependent NOX2 deficiency enhanced the macrophage maturation and inflammatory cytokine response; and provided help for CD4^+^ T cell activation in the context to *T. cruzi* infection in p47^phox−/−^ mice. Yet, early splenic response to *T. cruzi* infection (7 days pi) in p47^phox−/−^ mice was dominated by type 2 cytokines evidenced by a >2-fold decline in splenic TNF-α production and ∼2-fold increase in IL-10 release when compared to that noted in infected/WT controls, and likely responsible for susceptibility to *T. cruzi* infection (Fig. 5).

The phenotypic and functional characterization of CD8^+^T cells in p47^phox−/−^ mice provides clues to the cellular mechanisms contributing to increased susceptibility to *T. cruzi* infection. It was intriguing to find that splenocytes from p47^phox−/−^ mice, as compared to WT controls, contained ∼40% lower number of naïve CD8^+^ T cells. Further, CD8^+^T cells in p47^phox−/−^ mice exhibited no proliferation and activation evidenced by none-to-minimal increase in cell frequency overall or the frequency of IFNγ^+^, CD107^+^ or IFNγ^+^CD107^+^ CD8^+^T cells in response to *T. cruzi* infection and secondary *in vitro* stimulation with antigenic lysate (Fig. 6). Others have shown that T∶ B cell ratio is lower in p47^phox−/−^ mice as compared to the WT mice [30] and the CD8^+^T cells from p47^phox−/−^ mice express higher levels of pro-apoptotic Bim and Puma proteins that promoted their removal by apoptosis [31]. Since FOXO3 dephosphorylation (activation) by protein phosphatase 2A (PP2A) is known to contribute to transcriptional control of various apoptosis factors including pro-apoptotic Bim, blocking the PP2A activity attenuated the FOXO3 activation and Bim transcription and prolonged the survival of CD8^+^T lymphocytes in p47^phox−/−^ mice [31]. These studies suggest that p47phox deficiency adversely affects the development and survival of naive CD8^+^T cells. Additionally, treatment with apocynin that suppresses ROS production by NOX2 directly inhibited the production of proinflammatory cytokines (e.g. TNF-α, IFN-γ, and IL-2) in anti-CD3/anti-CD28-stimulated CD8^+^T cells. It is proposed that apocynin effects were mediated via attenuation of anti-CD3/anti-CD28-induced NF-κB activation in CD8^+^T cells [32]. The compromised CD8^+^T cell activation was not likely due to inefficient antigen presentation as p47^phox−/−^ dendritic cells are shown to be highly efficient in presentation of antigen to B cells in the context of antibody response to *Streptococcus* and *Listeria* infection [33]. Others have shown p47^phox−/−^ DCs elicit enhanced ovalbumin-specific CD4^+^ T lymphocytes [28]. Further studies will be required to delineate the complex role of p47^phox^ in antigen presentation by DCs, CD8^+^T lymphocytes survival and ROS-dependent mechanisms involved in NF-κB activation in cell-dependent manner. However, the literature discussed above and our findings allow us to surmise that compromised development of splenic CD8^+^T cells and their inability to respond to antigenic stimulus by generation of IFN-γ and cytolytic activity contributed to high tissue parasite burden in p47^phox−/−^ mice.

It is important to note that the components of NADPH oxidase have diverse effects in heart failure. For example, the survival rate of p47^phox−/−^ mice 4-weeks after myocardial infarction (MI) was significantly higher than that of WT mice (72% versus 48%) and the survival benefits were associated with a decline in LV dilatation and dysfunction, cardiomyocyte hypertrophy, apoptosis, and interstitial fibrosis in p47*^phox^* ^−/−^ mice [34]. Others have suggested the loss of p47^phox^ enhanced the susceptibility to heart failure. Patel et al [35] showed that the expression of N-cadherin and β-catenin was up regulated in p47^phox−/−^ mice subjected to biomechanical stress; however, actin filament cytoskeleton was disrupted because these mice lacked the ability to induce p47^phox^ dependent cortactin-N-cadherin interaction required for adaptive cytoskeletal remodeling. In comparison, gp91^phox−/−^ mice exhibited no increase in susceptibility to pressure overload and were equally capable of adaptive cytoskeletal modeling as was noted in controls. In the context of *T. cruzi* infection, Santiago et al [26] showed that gp91^phox−/−^ mice develop increased circulatory collapse and succumbed to infection. Authors proposed that while a lack of superoxide from phagocytes was not detrimental in hosts' ability to control parasites, superoxide regulates nitric oxide concentrations, and enhanced nitric oxide levels in these mice resulted in a critical drop in blood pressure. These studies suggest that targeting NADPH oxidase system as a potential novel therapeutic target to prevent cardiac failure should be considered with caution.

In summary, we present the first evidence that NOX2/ROS of macrophage origin shapes the T cell-mediated adaptive immunity, and its deficiency results in compromised CD8^+^T cell response to *T. cruzi* infection. Our data show that macrophages from p47^phox−/−^ mice were not compromised in the phagocytic activity and showed an enhanced iNOS/nitric oxide and pro-inflammatory cytokine levels in response to *T. cruzi* infection. However, in the event of NOX2 deficiency, generation and activation of CD8^+^T cell response was compromised, leading to increased parasite burden, tissue pathogenesis and mortality. We propose that future studies focused on understanding how NOX2/ROS induced innate receptor signaling pathways govern the activation and proliferation of T cell subsets will have the potential to identify specific targets for modulating the adaptive immunity and prevent *T. cruzi* infection and persistence in Chagas disease.

## Materials and Methods

### Ethics statement

All animal experiments were conducted following NIH guidelines for housing and care of laboratory animals and in accordance with The University of Texas Medical Branch at Galveston in accordance with protocols approved by the institution's Institutional Animal Care and Use Committee (protocol number 08-05-029).

### Parasites and mice

*T. cruzi* trypomastigotes (SylvioX10/4 strain) were maintained and propagated by continuous *in vitro* passage in C2C12 cells. C57BL/6 mice (WT and p47^phox−/−^) were purchased from Jackson Laboratory (Sacramento, CA). Mice (8-weeks-old) were infected with *T. cruzi* (2,000 or 10,000 trypomastigotes/mouse, intra-peritoneal). Survival from infection was monitored daily. Mice were sacrificed at day 7, 14, 21, and 30 post-infection (pi), and sera/plasma and tissue samples were stored at 4°C and −80°C, respectively.

### Western blotting

Bone marrow (BM) and splenic monocytes/macrophages, and heart and skeletal tissue were washed with ice-cold Tris-buffered saline (TBS), and homogenized in lysis buffer (tissue∶ buffer ratio, 1∶10, w/v). Homogenates (30-µg protein) were resolved on denaturing 10% acrylamide gels. Proteins were transferred to PVDF membranes, and probed with anti-p47^phox^ primary antibody (1∶1000, Santa Cruz, Dallas TX) for 24 h at 4°C. Membranes were washed with TBS containing 0.1% Tween-20 and TBS, incubated with horseradish peroxidase (HRP)-conjugated secondary antibody, and signal was developed by using a chemiluminiscence detection system (GE-Healthcare, Piscataway, NJ) [36].

### Tissue parasite burden

Skeletal muscle and heart tissues (50 mg) were subjected to Proteinase K lysis, and total DNA purified by phenol/chloroform extraction and ethanol precipitation method. Total DNA (100 ng) was used as a template in a PCR reaction for 28 cycles with oligonucleotides specific for *T. cruzi* 18S rDNA sequence (Forward: 5′-TAGTCATATGCTTGTTTC-3′, Reverse: 5′-GCAACAGCATTAATATACGC-3′) [19]. Quantitative estimate of parasite burden was obtained by real-time PCR on an iCycler thermal cycler with SYBR Green Supermix and *Tc18S*-sepecific oligonucleotides. Fold change was calculated as 2^−ΔCt^, where Δ^Ct^ represents the Ct (infected sample) - Ct (control) [20]. All data were normalized with host-specific GAPDH.

### RT-PCR

Total RNA was isolated by using the RNeasy plus Kit (Qiagen), and analyzed for quality and quantity on a SpectraMax UV microplate reader. After reverse transcription of 2 µg RNA with poly(dT)18, first-strand cDNA was used as a template in a real-time PCR on an iCycler Thermal Cycler with SYBR-Green Supermix (Bio-Rad) and specific oligonucleotides for *iNOS* (5′-GTTTCTGGCAGCAGCGGCTC-3′ and 5′-GCTCCfTCGCTCAAGTTCAGC-3′) and *GAPDH* (5′-TGG CAA AGT GGA GAT TGT TG-3′ and 5′-TTC AGC TCT GGG ATG ACC TT-3′). The PCR Base Line Subtracted Curve Fit mode was applied for Threshold Cycle (Ct) and mRNA level measured by iCycler iQ Real-Time Detection Software (Bio-Rad). The threshold cycle (*Ct*) values for target mRNA were normalized to *GAPDH* mRNA, and the relative expression level of *iNOS* was calculated with the formula *n*-fold change = 2−Δ*Ct*, where Δ*Ct* represents *Ct* (iNOS) – *Ct* (GAPDH) [37].

### Histology

Tissue sections were fixed in 10% buffered formalin for 24 h, dehydrated in absolute ethanol, cleared in xylene, and embedded in paraffin. Five-micron tissue-sections were stained with hematoxylin and eosin, and evaluated by light microscopy using an Olympus BX-15 microscope equipped with a digital camera. In general, we analyzed each tissue-section for >10-microscopic fields (100× magnification), and examined three different tissue sections/mouse (4 mice/group) to obtain a semi-quantitative score of parasitic pseudocysts (foci). Myocarditis (presence of inflammatory cells) was scored as 0 (absent), 1 (focal or mild with ≤1 foci), 2 (moderate with ≥2 inflammatory foci), 3 (extensive with generalized coalescing of inflammatory foci or disseminated inflammation), and 4 (diffused inflammation with severe tissue necrosis, interstitial edema, and loss of integrity) [38]. Inflammatory infiltrates was characterized as diffused or focal depending upon how closely the inflammatory cells were associated [39].

### Parasite uptake, intracellular replication, and release by macrophages

Splenic and BM monocytes from WT and p47^phox−/−^ mice were isolated as described [39]. Monocytes were distributed in 24-well plates (10^5^/well) incubated with *T. cruzi* trypomastigotes (live or heat-inactivated; cell: parasite ratio, 1∶3) for 0, 6, 12, and 24 h at 37°C, 5% CO_2_. In some experiments, monocytes were incubated with 5-µg/ml IFN-γ for 4 h before exposure to *T. cruzi*. Cells were submitted to Giemsa staining (Sigma-Aldrich, St. Louis, MO), and parasite uptake and intracellular replication monitored in 200 randomly selected cells by light microscopy.

*T. cruzi* trypomastigotes were labeled with 5-µM carboxyfluorescein succinimidyl ester (CFSE) fluorescent dye for 10 min, washed, and then incubated with splenocytes or BM monocytes (10^6^ cells/well; cell: parasite ratio, 1∶3) for 4 h. Cells were labeled with AP-anti-CD11b (macrophage marker) or R-PE-anti-Ly6G (neutrophil marker) antibodies (0.5-µg/100-µl, e-Biosciences, San Diego, CA), fixed with 2% paraformaldehyde, and re-suspended in 100-µl PBS/1% BSA, and analyzed by flow cytometry.

Trypomastigotes release in supernatants of *T. cruzi*-infected macrophages after 24 h and 48 h incubation was counted under a light microscope by using a hemacytometer.

### ROS production

Isolated primary monocytes from WT and p47^phox−/−^ mice were *in vitro* exposed to *T. cruzi* for 0–24 h as above. Cells were incubated for 30 min with 5-µM CM-H_2_DCF-DA (detects intracellular ROS, Ex_498 nm_/Em_598 nm_) in Hank's Balanced Salt Solution (HBSS), and signal was monitored on a SpectraMax M5 microplate reader (Molecular Devices, Sunnyvale, CA). In some experiments, isolated primary monocytes from WT and p47^phox−/−^ mice were *in vitro* exposed to *T. cruzi* for 24 h, and then incubated for 30 min with 0.1% nitroblue tetrazolium (NBT). NBT is a yellow water-soluble nitro-substituted aromatic tetrazolium compound that reacts with cellular superoxide ions to form water insoluble blue formazan crystals. Cells were counter-stained with safranin, and the percentage of NBT^+^ cells monitored by monitoring >200 randomly selected cells by light microscopy.

Mice (WT and p47^phox−/−^) were harvested at day 7–30 pi, and single cell suspension of splenic and BM cells were depleted of red blood cells by hypotonic lysis. Cells were cyto-spinned on glass slides (10^4^ cells/slide), equilibrated in Kreb's buffer, and incubated with 5-µM dihydroethidium (DHE, detects intracellular ROS, Ex_518 nm_/Em_605 nm_) and images captured by fluorescence microscopy [40]. Cells stained with DAPI (stains all nuclei, blue) were used as controls. Splenocytes (10^6^-cells/well/50 µl) were also incubated with APC-conjugated anti-CD11b antibody (e-Biosciences) and DHE, and macrophage-specific ROS production monitored by flow cytometry. All assays for monitoring the DCF or DHE fluorescence, NBT-based formazan crystal formation were performed in the presence and absence of 0.5 mM apocynin (NADPH oxidase inhibitor) to confirm the source of ROS.

### Nitric oxide levels

Splenocytes of *T. cruzi*-infected mice (10^6^-cells/well/100 µl) were incubated in presence or absence of *T. cruzi* antigenic lysate (TcL, 25-µg protein/well) for 24 h. TcL was prepared by subjecting parasites (1×10^9^/ml PBS) to 5–6 freeze-thaw cycles followed by sonication on ice for 30-min. Cells were stained with 5-µM DAF-FM (detects intracellular nitric oxide), and florescence (Ex_495 nm_/Em_515 nm_) was monitored on a SpectraMax M5 microplate reader [41].

Nitrite level in supernatants of splenocytes, *in vitro* stimulated in presence or absence of TcL, was measured by Griess reagent assay. Briefly, supernatants were reduced with 0.01 unit/100 ml of nitrate reductase, and incubated for 10 min with 100 ml of 1% sulfanilamide made in 5% phosphoric acid/0.1% N-(1-napthyl) ethylenediamine dihydrochloride (1∶1,v/v). Formation of diazonium salt was monitored at 545 nm (standard curve: 2–50 mM sodium nitrite). DAF-FM fluorescence and Griess reagent assays were performed in presence and absence of 5 µmol/ml N(G)-nitro-L-arginine methyl ester (L-NAME) that is an inhibitor of nitric oxide synthase [42].

### Cytokine release

Isolated primary splenocytes or BM monocytes were *in vitro* incubated with *T. cruzi* (live or heat-inactivated) for 48 h. The release of cytokines (IFN-γ, TNF-α, IL-4, IL-10) in cell free supernatants was determined by using optEIA™ ELISA kits, according to the manufacturer's specifications (BD Biosciences (San Jose, CA).

For estimating splenic production of cytokines, infected mice were harvested at day 7, 14, 21 and 30 pi, and single cell suspension of splenocytes (10^6^-cells/well/100 µl) incubated with media for 48 h (±TcL). Cytokine release was measured by an ELISA, as above.

### Lymphocytes' proliferation, intracellular cytokine response and cytotoxicity

Single-cell splenocytes from WT and p47^phox−/−^ mice harvested at day 30 pi were suspended in RPMI-5% FBS and distributed in 24-well plates (10^6^ cells/well/200 µl). Cells were incubated in presence of Con A (5 µg/ml), or *T cruzi* trypomastigote lysate (TcL, 25-µg/ml) at 37°C, 5% CO_2_ for 48 h. The cell suspensions were utilized to measure the T cell proliferation by MTT assay [43].

To identify the T cell subsets in infected mice, splenocytes were incubated with or without (TcL), and then labeled for 30 min on ice with PE-Cy7-anti-CD3 (binds all T cells), FITC-anti-CD8 and PE-anti-CD4 antibodies (0.5–1 µg/100 µl, e-Biosciences). Following incubation, cells were fixed, washed and re-suspended in 100 µl PBS/2% BSA, and analyzed by flow cytometry [44].

To monitor the intracellular cytokine response, splenocytes were *in vitro* stimulated as above except that brefeldin A (10-µg/ml; Sigma) was added in the final 6 h to prevent protein secretion. Cells were labeled with PE-anti-CD4 and FITC-anti-CD8 antibodies, fixed, suspended in 100-µl permeabilization buffer (0.1% saponin/1% FBS in PBS) and then utilized for intracellular staining with e-Fluor-anti-IFN-γ, Cy5-anti-TNF-α and PerCP-PA-anti-Ki67 antibodies (0.5–2-µg/100-µl, e-Biosciences). Splenocytes were also incubated with Alexa-Fluor488-anti-CD107 antibody to determine the cytolytic activity of the activated/proliferating T cell subpopulations. Cells stained with isotype-matched IgGs were used as controls. Samples were visualized on a LSRII Fortessa Cell Analyzer by six-color flow cytometry, acquiring 30–50,000 events in a live lymphocyte gate, and further analysis performed using FlowJo software (ver.10.0.6, Tree-Star, San Carlo, CA) [44].

### Data analysis

Data (mean ± SD) were derived from at least triplicate observations per sample (*n*≥8 animals/group). Normally distributed data (confirmed by Histogram and Q-Q plots) were analyzed by student's t-test (comparison of 2-groups) and 1-way analysis of variance (ANOVA) with Tukey's post-hoc test (comparison of multiple groups). The level of significance is presented by ^*^ (normal-versus-infected) and ^#^ (WT-versus-p47^phox−/−^) (^*, #^ *p*<0.05, ^**,##^ *p*<0.01).

## Supporting Information

Figure S1 **Parasite uptake and replication in p47^phox−/−^ macrophages.** Primary splenic monocytes from WT and p47^phox−/−^ mice were isolated as described in Materials and Methods. Single cell population (10^5^-cells/well) was differentiated to macrophages in presence of 5 µg/ml IFNγ and then incubated with *T. cruzi* (1∶3, cell: parasite ratio). Cells were submitted to Giemsa staining, and parasite uptake and intracellular replication monitored in 200 randomly selected cells by light microscopy. Shown are representative images of WT (panels a–e) and p47^phox−/−^ (panels g–j) monocytes that were stimulated with IFN-γ, and then incubated with *T. cruzi* for 0 (b&g), 6 (c&h), 12 (d&i), and 24 (e&j) h. Unstimulated WT and p47^phox−/−^ splenocytes are shown in panels a and f, respectively.
(TIF)

Figure S2 **Tissue analysis of inflammatory infiltrate in** ***T. cruzi*** **-infected p47^phox−/−^ mice.** C57BL6 WT (10000 parasites/mouse) and p47^phox−/−^ (2000 parasites/mouse) mice were intraperitoneally injected with *T. cruzi*. Shown are representative images of H&E staining (blue: nuclear, pink: muscle/cytoplasm/keratin) of skeletal muscle (panels a–e) and heart tissue (panels f–o) sections from WT (panels a–j) and p47^phox−/−^ (panels k–o) mice harvested at day 0 (a,f,k), 7 (b,g,i), 14 (c,h,m), 21 (d,i,n), and 30 (e,j,o) post-infection (magnification: 20×). H&E staining of tissue sections from WT mice infected with 2000 parasites are presented in Fig. 4 in the main text.
(TIF)
