# Supplementary figures and images for: P47phox−/− Mice Are Compromised in Expansion and Activation of CD8+ T Cells and Susceptible to Trypanosoma cruzi Infection
Source: PLoS Pathog. 2014 Dec 4;10(12):e1004516. doi: 10.1371/journal.ppat.1004516 (PMC4256457; doi:10.1371/journal.ppat.1004516)

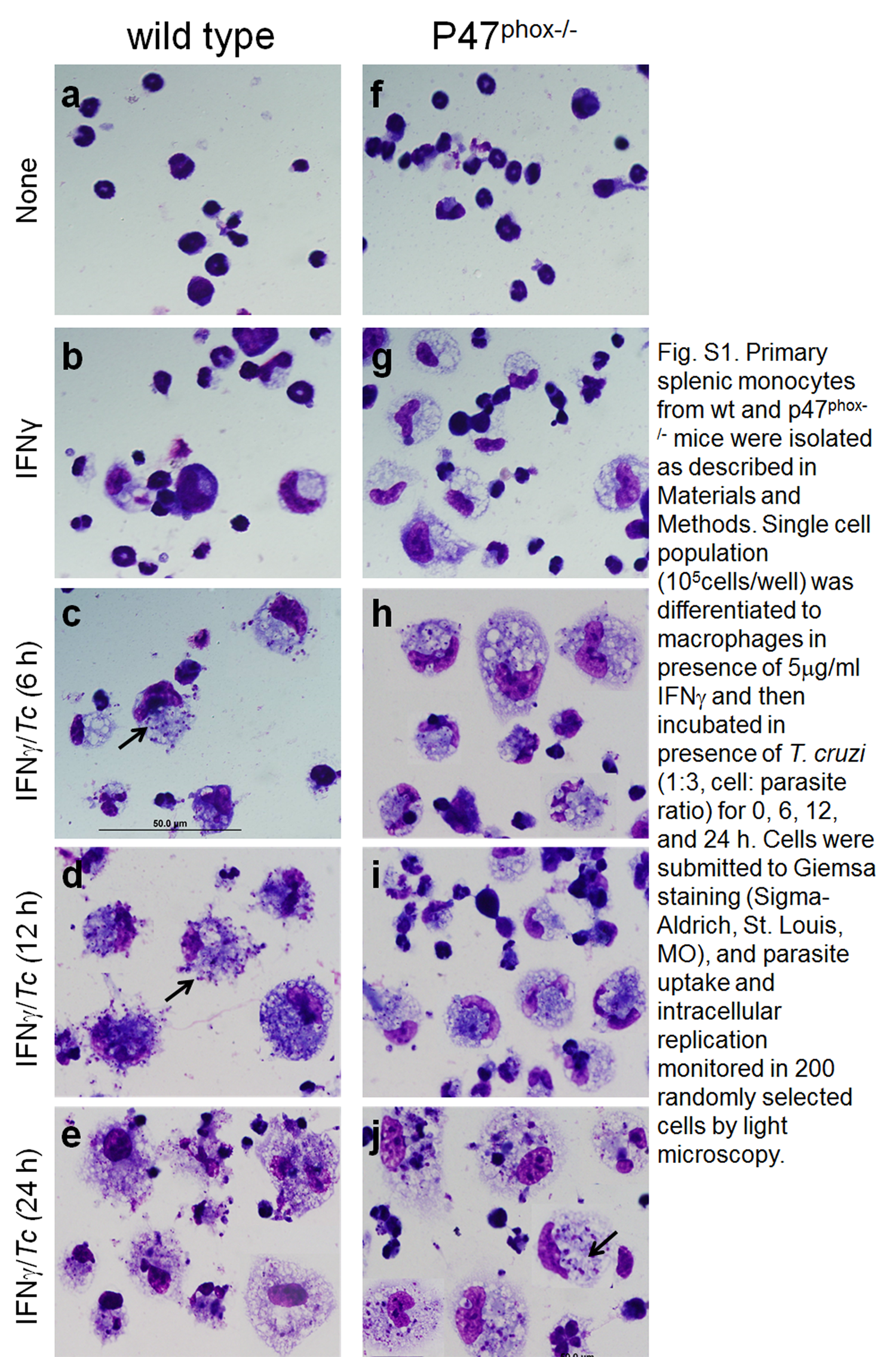

Supplement: Figure S1 — Parasite uptake and replication in p47phox−/− macrophages. Primary splenic monocytes from WT and p47phox−/− mice were isolated as described in Materials and Methods. Single cell population (105-cells/well) was differentiated to macrophages in presence of 5 µg/ml IFNγ and then incubated with T. cruzi (1∶3, cell: parasite ratio). Cells were submitted to Giemsa staining, and parasite uptake and intracellular replication monitored in 200 randomly selected cells by light microscopy. Shown are representative images of WT (panels a–e) and p47phox−/− (panels g–j) monocytes that were stimulated with IFN-γ, and then incubated with T. cruzi for 0 (b&g), 6 (c&h), 12 (d&i), and 24 (e&j) h. Unstimulated WT and p47phox−/− splenocytes are shown in panels a and f, respectively. (TIF) [file ppat.1004516.s001.tif]

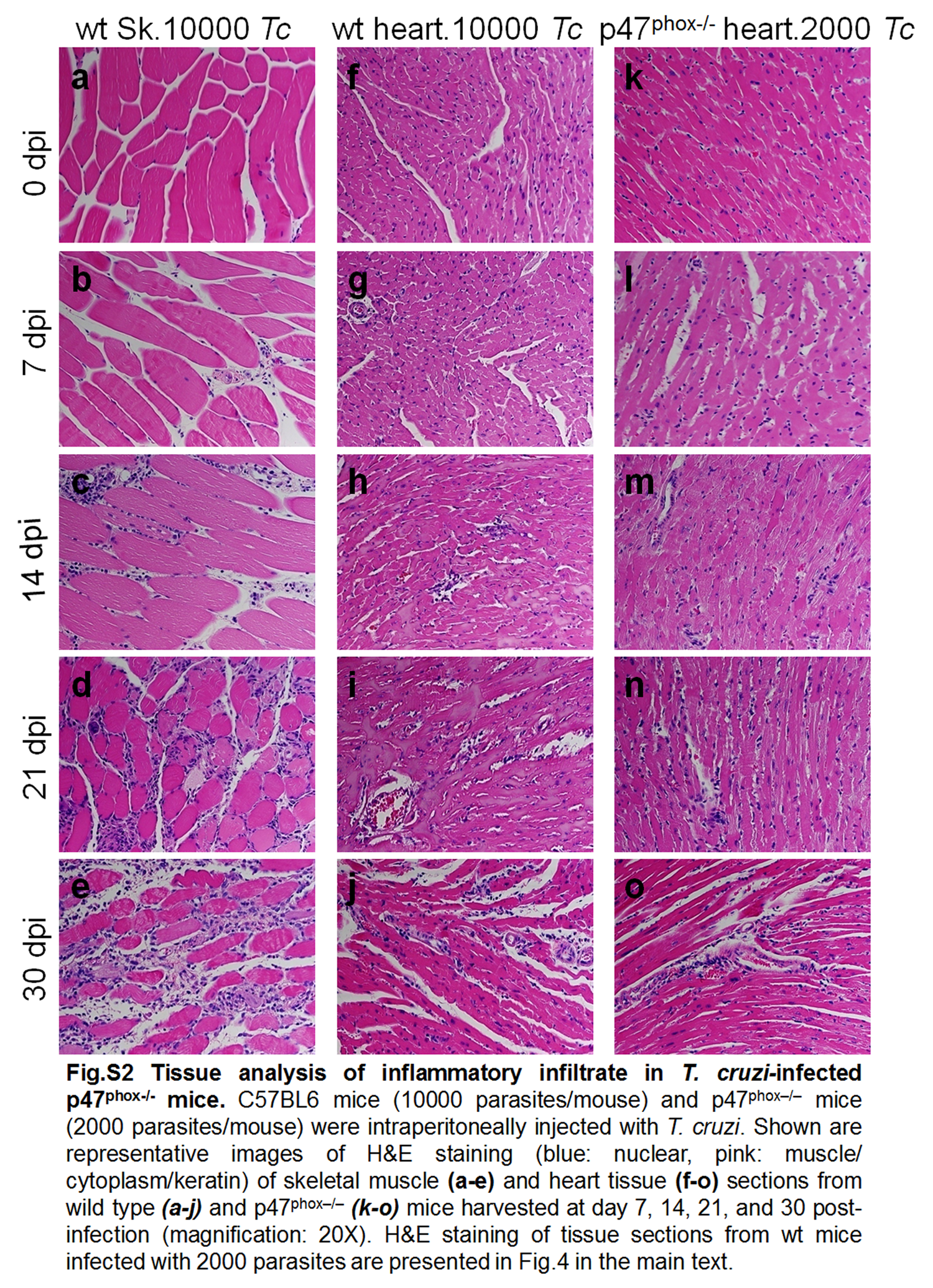

Supplement: Figure S2 — Tissue analysis of inflammatory infiltrate in T. cruzi -infected p47phox−/− mice. C57BL6 WT (10000 parasites/mouse) and p47phox−/− (2000 parasites/mouse) mice were intraperitoneally injected with T. cruzi. Shown are representative images of H&E staining (blue: nuclear, pink: muscle/cytoplasm/keratin) of skeletal muscle (panels a–e) and heart tissue (panels f–o) sections from WT (panels a–j) and p47phox−/− (panels k–o) mice harvested at day 0 (a,f,k), 7 (b,g,i), 14 (c,h,m), 21 (d,i,n), and 30 (e,j,o) post-infection (magnification: 20×). H&E staining of tissue sections from WT mice infected with 2000 parasites are presented in Fig. 4 in the main text. (TIF) [file ppat.1004516.s002.tif]
